# Spinal Anaesthesia for Urological Surgery: A Comparison of Isobaric Solutions of Levobupivacaine and Ropivacaine with Dexmedetomidine

**DOI:** 10.4314/ejhs.v33i1.9

**Published:** 2023-01

**Authors:** Bhagyesh Sushchendra Kame, Vaishali U Kumar, Anand Subramaniam

**Affiliations:** 1 Department of Anaesthesiology, Chettinad Hospital and Research Institute, Tamil Nadu, India

**Keywords:** Isobaric Ropivacaine, Isobaric Levobupivacaine, Dexmedetomidine, Subarachnoid Block, Urological surgeries, Spinal Anaesthesia

## Abstract

**Background:**

Subarachnoid block is used in most of urological surgeries and finding the best possible drug has always been a challenge. Bupivacaine's pure enantiomers ropivacaine and levobupivacaine have lesser systemic toxicity. Isobaric solution has extra benefit of not affecting the intrathecal dispersion of drug. Dexmedetomidine when added intrathecally provides longer duration of analgesia and anaesthesia. Aim of this study is to compare onset, duration of the block with both the drugs along with their hemostability and postoperative analgesia.

**Methods:**

It is a Prospective Randomized Double-Blind Study. It includes 68 patients undergoing urological procedures under subarachnoid block. Group LD: Patients will receive 3.5 ml of Isobaric Levobupivacaine 0.5% + Dexmedetomidine 10 µg (0.1ml) Group RD: will receive 3.5ml of Isobaric Ropivacaine 0.5% + Dexmedetomidine 10 µg (0.1ml)

**Results:**

Time taken for onset of sensory and motor block is significantly more in ropivacaine while duration of block is more in levobupivacaine.

**Conclusions:**

Addition of Dexmedetomidine to Isobaric Levobupivacaine significantly prolongs the duration of analgesia and anaesthesia compared to Ropivacaine and maintains stable hemodynamics. Ropivacaine is a suitable drug for day care whilst levobupivacaine is an excellent agent for longer surgeries. Dexmedetomidine is an effective non-opioid adjuvant which improves effectiveness of block without increasing the risk of side effects.

## Introduction

Spinal anaesthesia due to the sheer benefits of an awake patient, low drug costs, excellent intraoperative anaesthesia, prolonged and satisfactory postoperative analgesia, and quick patient turnover, seems to have become the preferred method of choice before general anaesthesia for lower abdominal, lower limb, pelvic, and perineum surgeries (1).

Bupivacaine is the first amide-linked long-acting local anaesthetic that has edge of having longer duration of action than lignocaine. In the recent years, it's pure enantiomers such as ropivacaine [amides] and levobupivacaine [amides], because of their decreased toxicity for the cardiovascular and central nervous systems, have been incorporated into clinical practice. Isobaric solutions have the extra benefit of not affecting the intrathecal dispersion of local anaesthetic during and after injection (2).

The S (-) enantiomer of racemic bupivacaine, levobupivacaine, is less toxic to the heart and central nervous system than racemic bupivacaine (2). Reports of levobupivacaine being used for epidural or brachial plexus anaesthesia suggested that it had the same clinical efficacy as Bupivacaine (3). In subarachnoid block, levobupivacaine has similar effects to bupivacaine, and motor block reversion occurs early (4). It causes differential neuraxial blockade with motor function preservation at low concentrations, making it suitable for ambulatory surgery (5). Ropivacaine is pure S-enantiomer of bupivacaine. Compared to other local anesthetics, ropivacaine has less motor blockade, cardiovascular and neurological toxicity, also produces differential neural blockade (6). Due to the low prevalence of transient neurological symptoms, it is an alternative to ambulatory lidocaine surgery (7). Although ropivacaine has a Pka that is similar to bupivacaine, it is less fat-soluble, it should block A-alpha fibres more slowly than bupivacaine (6). Ropivacaine, as opposed to bupivacaine, has been shown to induce less severe motor blockade. It may be helpful in subarachnoid blockade considering of its brief timeframe, faster recovery of motor function plus lesser toxicity profile (8).

Many additives to LA have been utilised intrathecally to enhance the quality of intraoperative analgesia and extend it in the postoperative period (9). To achieve this, opioids such as morphine, buprenorphine, pethidine, hydromorphone, fentanyl, sufentanil, tramadol and alpha-2 adrenergic agonists like dexmedetomidine, clonidine have been supplemented in spinal local anaesthetics (10,11). Clonidine and dexmedetomidine, intrathecal alpha-2 agonists, are utilized as neuraxial adjuvant agents because they do not cause pruritis or respiratory depression like spinal opioids do. They enhance the efficacy of local anesthetics and enable for lower doses to be used. Clonidine, an intrathecal Alpha-2 adrenoreceptor agonist, has been the subject of the majority of clinical trials. There are few data on the efficacy of dexmedetomidine as a neuraxial adjuvant. Dexmedetomidine is an alpha-2 adrenoreceptor agonist that has been recognized for use as an intravenous sedative and co-analgesic drug. It has a selectivity ratio of alpha-2/alpha-1 that is eight times higher than clonidine (12). Previous clinical trials have shown that intravenous dexmedetomidine has a strong opioid sparing effect and reduces the need for inhalational anesthetics (12).

**Primary objective** of our study is to compare onset and duration of sensory and motor blockade. **Secondary objectives** are to assess and compare the hemodynamic between both groups to assess two segment sensory regression time and to identify side effects, if any.

## Materials and Methods

**Study design**: It is a prospective, randomized, double blinded study carried out at Chettinad Hospital and Research Institute in Chennai, under the Department of Anaesthesiology.

**Study population**: Patients scheduled for urological surgeries under subarachnoid block who meet the inclusion criteria. The Institutional Human Ethical Committee reviewed and approved the study - IHEC No: 024/IHEC/Jan 2021, dated 02.02.2021, CTRI/2021/08/035681. Prior to enrolment all study participants were explained the risks and benefits associated with the study in a language they understand, following which an informed written consent was obtained. We selected 68 patients and randomly divided them into two groups (34 patients in each group) using a computer-generated randomization code. Study was done from February 2021 to September 2021.

While American Society of Anaesthesiologist (ASA) grade I-II, age group between 18–65 years and scheduled for urology surgery were the inclusion criteria; patient refusal (not willing for regional anaesthesia), patients on (alpha adrenergic receptors antagonists, Ca2+ channel blockers, ACE inhibitors), history of allergy to study drugs, post-spinal surgeries, spinal deformities, coagulopathy, dysrhythmia, major dysfunctions (hepatic, renal and cardiovascular), BMI >35 and Height <140cm were the exclusion criteria.

All the patients had regular pre-operative evaluations at the pre-anesthetic assessment clinic and were assessed again the day before surgery. Prior to surgery, all patients are recommended to stay nil per oral: 8 hours for solid meal, and 4 hours for oral clear liquids. They were also informed about the benefits and drawbacks of spinal anesthesia. They were given Tab. Ranitidine 150mg the night before surgery and 6AM on the day of operation. On arrival to the pre-anaesthetic receiving area, the consent forms were rechecked, an 18G IV access secured and the patients were preloaded with 10–15 ml/kg ringer lactate 15 minutes preceding to surgery. Once the patient was shifted to the operating room, routine monitors for hemodynamic monitoring (3- lead ECG monitoring, heart rate, blood pressure and oxygen saturation) were attached and baseline vital signs were recorded.

For Spinal Anaesthesia, patient was put in sitting position, under aseptic precautions L3-L4 inter-space infiltrated with 2ml of 2% Inj. Lignocaine. Double blinding was done using a computer-generated code. The coding sheet was given to an individual who was not participating in the study to prepare the study drug. Group LD: Patients will receive 3.5 ml of Isobaric Levobupivacaine 0.5% + Dexmedetomidine 10 µg (0.1ml) and Group RD: will receive 3.5ml of Isobaric Ropivacaine 0.5% + Dexmedetomidine 10 µg (0.1ml). A 26-Gauge Quincke spinal needle was used. The proper needle insertion was identified by free flow of cerebrospinal fluid, after which anaesthesiologist administered 3.6 ml of study drug. The patient was immediately put in supine position to conduct the initial assessment.

The onset of sensory and motor blockade was assessed at baseline and 3 min interval up to 15 min thereafter 5 mins interval up to 30 minutes. The patient's heart rate, blood pressure, and saturation were measured every 3 mins interval for the 1st 15 min, then every 5 mins interval for the next 60 min, and thereafter every 10 mins until operation was completed. All patients were given 6 liters of oxygen per minute through a facemask throughout the surgery.

**Sensory block**: Loss of pinprick feeling was used to measure the degree of sensory block. The dermatomes S1, L3, T12, T10, T8, T6 and upper T4 were examined bilaterally. The C5-C6 area was utilized as a reference point for normal feeling. Sensory onset was defined as the absence of a pin prick feeling with a 23G needle at the T10 level. The test was repeated every 3 mins interval until 15 mins, thereafter every 5 mins until 30 min. 31 The duration of sensory blockade was defined as the time elapsed between the injection of a drug intrathecally and the point at which the sensory block was completely resolved when the patient requested analgesia for post-operative pain (VAS > 3). The time required for the block's two segment regressions was also recorded.

**Motor Block**: The time required to attain a motor block of Grade 2, as graded by the Modified Bromage Score used by Saxena et al (13), was defined as the onset of the motor block. Using the Modified Bromage Score, the duration of motor block was defined as the time elapsed between the start of a complete motor block (Grade 3) and the patient's ability to bend the knees (Grade 0). The motor regression time was measured using Modified Bromage Score. If the subarachnoid block proved ineffective, general anaesthesia was administered, and the patient was excluded from the analysis

**Vital signs and side effects**: The patient's heart rate, blood pressure, and SPO2 are measured every 3 mins interval first 15 mins, then every 5 mins interval for next 60 mins and thereafter every 10 mins until operation is completed.

**Hypotension:** A drop in systolic blood pressure of less than 90 mmHg or drop in blood pressure more than 30 mmHg from baseline was classified as hypotension. The patient was treated with 6mg increments of intravenous ephedrine.

**Bradycardia:** Heart rate less than 50/min was considered as Bradycardia and treated by giving incremental doses of 0.3mg intravenous Atropine.

**Respiratory Depression:** A respiratory rate of less than 8 breaths per minute and/or a SpO2 of less than 90% were considered to be signs of respiratory depression. In case of respiratory depression,100% oxygen was administered to the patient via bag and mask ventilation.

The time lapse between the delivery of spinal anaesthesia and the request for first rescue analgesia was recorded. Patients were considered to have significant pain if they recorded VAS score >/= 3 at rest and a VAS score of >/=5 with movement. In such cases of pain Inj. Tramadol 50mg was given intravenously and Inj. Ondansetron 4mg in cases experiencing nausea and vomiting. Postoperatively patients monitored for changes in vital parameters, nausea, vomiting, shivering, respiratory depression, urinary retention and treated appropriately

Descriptive analysis was carried out by mean and standard deviation for quantitative variables, frequency and proportion for categorical variables. Data was also represented using appropriate diagrams like bar diagram, pie diagram. For normally distributed Quantitative parameters the mean values were compared between study groups using independent sample t-test {2 groups}. Categorical outcomes were compared between study groups using Chi square test /Fisher's Exact test {If the overall sample size was < 20 or if the expected number in any one of the cells is < 5, Fisher's exact test was used}. P value < 0.05 was considered statistically significant. IBM Corp. Released 2013. IBM SPSS Statistics for Windows, Version 22.0. Armonk, NY: IBM Corp. was used for statistical analysis

## Results

There is a significant difference in Onset of Sensory Block {T10} between study group. {P value < 0.001}. There is a significant difference in Time for onset of motor block between study group. {P value 0.016}. The mean time for complete block {in minutes} was 8.03 ± 2.53 in Group LD and it was 9.18 ± 1.8 in Group RD. There is a significant difference in time for complete block {in minutes} between study groups {P value 0.035}. There is a significant difference in time taken to achieve T8 and T6 {in minutes} between study groups {P value < 0.05}. There is a significant difference in Duration of motor Blockade between study groups {P value < 0.001}. There is a significant difference in Time of two segment regression from highest sensory level between study groups {P value < 0.001}. There is a significant difference in Regression of Sensory level {up to T10} between study groups {P value 0.002}. There is a significant difference in Time for First Rescue Analgesia between study groups {P value < 0.001}.

## Discussion

Bupivacaine is the most powerful local anaesthetic comparable to levobupivacaine, followed by ropivacaine, according to clinical trials in diverse patient groups (14). Ropivacaine is less powerful because of its reduced lipid solubility, but it has the benefit of better sensory and motor block differentiation, which is especially beneficial when early ambulation is required to improve recovery (15).

Although randomized controlled trials have shown that ropivacaine and levobupivacaine are efficacious at producing analgesia and anaesthesia when used in lower extremity surgeries, there is little documentation about their clinical profiles (15,14). Because of its quick initiation and longer duration of sensory block, shorter duration of motor block, and reduced cardiac toxicity, levobupivacaine is a preferable local anaesthetic when compared to bupivacaine (16). Previous research indicated that combining dexmedetomidine with levobupivacaine generates efficient analgesia, extends duration of motor and sensory block, and offer superior postoperative analgesia with less detrimental effects (17).

Although the mechanism is unknown, α2 adrenoceptor agonists have been shown to prolong the sensory and motor block durations of local anaesthesia. α2 adrenoceptors are found over the main afferent terminals of neurons in the superficial lamina of the spinal cord and in pain-related brainstem nuclei. This localization supports the notion that 2 agonists exert analgesic effects via both peripheral and central routes (18,19). Dexmedetomidine has emerged as a possible adjuvant with a facilitative impact on LA. Dexmedetomidine has been utilized as a supplement to local anaesthetics for peripheral nerve blocking in number of studies described in the literature. It has increased the efficacy of the block in most of them, with no evidence of neurological adverse effects (20). Motor and sensory block was significantly prolonged on addition of dexmedetomidine which provides for better patient compliance in the postoperative period (21). The use of isobaric solutions may prove less sensitive to position concerns, its baricity has the benefit of producing a less position sensitive block.

**Onset of sensory and motor block**: The mean time to start of sensory block in our present investigation was 4.32 ± 1.51 minutes in Group LD and 7.24 ± 1.5 minutes in Group RD. When comparing Ropivacaine to Levobupivacaine, the mean time for onset of sensory block {T10} was substantially longer in the Ropivacaine group. The mean time for the start of motor block in Group LD was 4.5 ± 1.85 minutes and 5.74 ± 2.26 minutes in Group RD, which was considerably longer than in Group RD. Jain S et al {2017} (15) undertook a study and surmised that time of onset of sensory block in levobupivacaine and ropivacaine group are {6.30±1.39; 8.23±2.84} respectively. Whereas onset of motor block took {5.33±2.19; 6.63±2.34} minutes in levobupivacaine and ropivacaine respectively. Al-Mustafa et al. (22) Surmised when 5 microgram and 10 microgram dexmedetomidine were administered to spinal bupivacaine it took 4.7 ± 2.0 minutes in D10 group while 6.3 ± 2.7 minutes in D5 group for commencement of sensory block{T10}.

**Time taken to achieve T8 and T6**: Time Taken to achieve T8 and Time taken to achieve T6 was significantly lesser among Group LD {7.41±3.64 and 11.71±4.25} respectively compared to Group RD {9.44±3.14 and 15.15±4.05} respectively. Athar et al (8) The median maximum height achieved in terms of dermatomes in both the groups was T_7_ {T_5_-T_10_} , however the time to reach maximum height was shorter in group R {13.17 ± 3.02 min} as compared to group L {20.33 ± 5.31 min} with a *p* < 0.0001, which is different from our study. Luck et al (7) showed no statistically significant difference in time taken to reach maximum cephalad spread between the groups.

**Time taken for complete motor blockade**: The mean Time taken for complete motor block was significantly more among Group RD {9.18 ± 1.8} compared to Group LD {8.03 ± 2.53}. Patel et al. {2018} (5) undertook a study on 68 patients who underwent lower limb surgery under spinal anaesthesia using 3ml of 0.5% levobupivacaine and 3 ml of 0.75% Ropivacaine. She surmised that it took {6.68 ± 1.147} minutes to reach Bromage scale grade 3 in Group L and {7.97 ± 0.87} minutes in Group R.

**Hemodynamic variables**: There was no remarkable statistical variation in Hemodynamic Variables at different follow up periods between study groups {P value >0.05}. In a study done by Athar et al (8), on Levobupivacaine or Ropivacaine based on Equipotent Doses in Spinal Anaesthesia where 3ml 0.5% levobupivacaine and 3ml 0.75% Ropivacaine was given, they reported that Levobupivacaine has longer duration of action but efficacy, toxicity and hemodynamic stability makes ropivacaine suitable for surgeries with low threshold hypotension which was partially related to our study because in our study no hemodynamic instability noted in both the study groups.

**Duration of motor blockade**: The mean duration of motor Blockade was significantly more among Group LD {393.53 ± 25.33} compared to Group RD {267.35 ± 23.78}. In similarity to current study, Luck et al (7) Reported that, the extent and duration of motor block were substantially lower in the ropivacaine group when compared to the bupivacaine and levobupivacaine groups. Athar et al (8) observed that Levobupivacaine caused a considerably longer duration of motor block {290.50 ± 34.67} than ropivacaine {222.50 ± 23.00} the findings were quite similar to the present study.

**Mean of time for first rescue analgesia**: The mean Time for First Rescue Analgesia in the Group LD was {290.29 ± 29.28} considerably higher than in the Group RD {197.65 ± 26.75}. Sriramka et al (23) observed that duration of analgesia was found to be slightly longer in group LD (955.3 ± 114.5 minutes) than in group RD patients (894.6 ± 91.3) with p = 0.027, with a higher rescue analgesia requirement in RD group.

**The mean time for two segment regressions of sensory block**: The mean of Time for 2 segment regressions from highest sensory level was considerably more in Group LD {115.59 ± 11.33} compared to Group RD {92.65 ± 11.09}

**Side effects**: The incidence of side effects among study groups such as nausea, bradycardia, vomiting, hypotension and shivering were comparable. There was no statistically significant difference in incidence of side effects among the study groups {P>0.05}.

We thereby conclude that the isobaric levobupivacaine and ropivacaine dosages employed in the trial provide sufficient anaesthesia and analgesia for operations while causing no significant adverse effects. Levobupivacaine causes considerably longer duration of analgesia than ropivacaine. Because of its efficacy, toxicity and hemodynamic profile ropivacaine is a suitable drug for day care and operations with a lower hypotension threshold whilst levobupivacaine is an excellent agent for longer surgeries. Dexmedetomidine improves effectiveness of intrathecal local anaesthetics without increasing the risk of side effects. It appears to be an appealing adjuvant

## Figures and Tables

**Table 1:** Comparison of mean of Onset of Sensory Block (T10), time taken to achieve sensory level of T8 and T6 and the Mean of Time of Two Segment Regression from Highest Sensory Level between study group (N=68)

PARAMETER	STUDY GROUP (mean + SD)		P value
		
	GROUP LD (n=34)	GROUP RD (n=34)	
Onset of sensory block (T10)	4.32+1.51	7.24+1.5	<0.001
Time taken to achieve T8(mins)	7.41+3.64	9.44+3.14	0.016
Time taken to achieve T6(mins)	11.71+4,25	15.15+4.05	0.001
Time of two segment regression from highest sensory level	115.59+11.33	92.65+11.09	<0.001

**Table 2:** Comparison of mean of time for onset and mean duration of motor block between study group (N=68)

PARAMETER	STUDY GROUPS (mean±SD)		P value
		
	GROUP LD (n=34)	GROUP RD (n=34)	
Time for onset of motor block	4.5=1.85	5.74+2.26	0.016
Duration of motor block	393.53+25.33	267.35+23.78	<0.001
